# Bereavement and Physiological Dysregulations in African American Adults

**DOI:** 10.1093/geroni/igaa057.3424

**Published:** 2020-12-16

**Authors:** Jieun Song, Marsha Mailick

**Affiliations:** 1 Waisman center, Madison, Wisconsin, United States; 2 University of Wisconsin - Madison, Madison, Wisconsin, United States

## Abstract

This study uses data from National Survey of Midlife in the U.S. (MIDUS) to examine the effect of bereavement on physiological dysregulations in African American adults, with moderating effects of gender. Models were estimated using data from 210 Non-Hispanic African American respondents who participated in MIDUS 2 (M2: 2004-2005) and the biomarker data collection (2004-2009). We analyzed data from two groups, respondents who experienced the death of an individual(s) close to them, either family or friends (97 women, 40 men) and respondents who did not experience any deaths of close individuals during the same period (46 women, 27 men), controlling for age, education, marital status, prior family bereavement, number of negative life events since M2, and physical health prior to bereavement. Physiological dysregulations were assessed for 7 systems: HPA axis, glucose metabolism, lipids metabolism, sympathetic system, parasympathetic system, inflammation, and cardiovascular functioning. The results show that African American men and women who experienced bereavement were at higher risk of dysregulation of glucose metabolism (assessed by HbA1c, HOMA-IR, and fasting glucose) than the non-bereaved, even after adjusting prior diabetes diagnosis. In addition, African American women (but not men) who experienced recent bereavement were at higher risk of dysregulation of HPA axis functioning (assessed by urinary cortisol and blood DHEA-S) than their counterparts. The other physiological systems were not significantly associated with bereavement experience in African American adults. The findings suggest that bereavement has adverse impacts on health in African American adults via dysregulations in glucose metabolism and HPA axis functioning.

